# DJ-1 regulates mitochondrial function and promotes retinal ganglion cell survival under high glucose-induced oxidative stress

**DOI:** 10.3389/fphar.2024.1455439

**Published:** 2024-09-11

**Authors:** Hanhan Peng, Haoyu Li, Benteng Ma, Xinyue Sun, Baihua Chen

**Affiliations:** ^1^ Department of Ophthalmology, The Second Xiangya Hospital, Central South University, Changsha, China; ^2^ Hunan Clinical Research Centre of Ophthalmic Disease, Changsha, China

**Keywords:** diabetic retinopathy, retinal ganglion cells, mitochondria, oxidative stress, DJ-1

## Abstract

**Purpose:**

This study aimed to investigate the antioxidative and neuroprotective effects of DJ-1 in mitigating retinal ganglion cell (RGC) damage induced by high glucose (HG).

**Methods:**

A diabetic mouse model and an HG-induced R28 cell model were employed for loss- and gain-of-function experiments. The expression levels of apoptosis and oxidative stress-related factors, including Bax, Bcl-2, caspase3, Catalase, MnSOD, GCLC, Cyto c, and GPx-1/2, were assessed in both animal and cell models using Western blotting. Retinal structure and function were evaluated through HE staining, electroretinogram, and RGC counting. Mitochondrial function and apoptosis were determined using JC-1 and TUNEL staining, and reactive oxygen species (ROS) measurement.

**Results:**

In the mouse model, hyperglycemia resulted in reduced retinal DJ-1 expression, retinal structural and functional damage, disrupted redox protein profiles, and mitochondrial dysfunction. Elevated glucose levels induced mitochondrial impairment, ROS generation, abnormal protein expression, and apoptosis in R28 cells. Augmenting DJ-1 expression demonstrated a restoration of mitochondrial homeostasis and alleviated diabetes-induced morphological and functional impairments both *in vivo* and *in vitro*.

**Conclusion:**

This study provides novel insights into the regulatory role of DJ-1 in mitochondrial dynamics, suggesting a potential avenue for enhancing RGC survival in diabetic retinopathy.

## 1 Introduction

Diabetes mellitus (DM) is a chronic metabolic and endocrine disorder arising from inadequate insulin secretion or resistance, presenting a substantial global public health challenge (Sun et al., 2022). Its high incidence and diverse complications, notably diabetic retinopathy (DR) (Leasher et al., 2016), spotlight its global impact. Traditionally characterized as dysfunction within retinal vasculature (Wong et al., 2016; GBD, 2019 Blindness and Vision Impairment Collaborators, 2021), DR has recently been associated with diabetic retinal neurodegeneration (DRN), which may precede and contribute to microangiopathy progression (Soni et al., 2021). The neurovascular unit (NVU) concept has elucidated the interplay between DRN and diabetic retinal vasculopathy (DRV) (Peng et al., 2022). Disturbances within the NVU, consisting of neurons, vascular cells, glial cells, and local immune cells, may underlie the early stages of DR.

The retina, an extension of the central nervous system, boasts a specialized multilayered structure rich in retinal ganglion cells (RGCs). As the sole neurons transmitting visual information from the retina to the brain, preserving the structural and functional integrity of RGCs is paramount for vision. The high metabolic activity of RGCs demands an abundance of mitochondria in their axons, resulting in elevated reactive oxygen species (ROS) production compared to general neurons (Ito and Di Polo, 2017). ROS, inevitable by-products of metabolism, are generated through various pathways, including the mitochondrial electron transport chain and cytochrome P450 (Wang et al., 2023). While defense mechanisms exist to maintain ROS balance (Zhu et al., 2023; Napolitano et al., 2021), oxidative stress arises from an imbalance between oxidative and antioxidant systems, leading to cytotoxic effects. Our previous studies highlighted oxidative stress as a crucial trigger for DR onset and progression, RGC apoptosis, and demonstrated that antioxidant therapy enhances mitochondrial function in RGCs and the optic nerve (Zhang et al., 2015; Zhang et al., 2019).

Crucially, DJ-1 serves as a significant regulator of ROS production (Dolgacheva et al., 2019; Amatullah et al., 2021). Under oxidative conditions, DJ-1 functions as a redox-sensitive chaperone and oxidative stress sensor, inhibiting α-synuclein aggregation in neurodegenerative diseases (Shendelman et al., 2004; Zhou et al., 2006). Additionally, DJ-1 exerts a neuroprotective role against oxidative stress in neurons and retinal pigment epithelium (Martin-Nieto et al., 2019; Shadrach et al., 2013; Upadhyay et al., 2020). Our previous research demonstrated that DJ-1 overexpression significantly improves mitochondrial function, reduces ROS production, and enhances antioxidant enzyme activity in retinal capillary pericytes induced by high glucose (HG), therefore regulating retinal capillary pericyte death in DR (Wang et al., 2020; Zeng et al., 2019). Therefore, we hypothesize whether DJ-1 possesses a neuroprotective effect involving the mentioned mechanism, warranting further investigation.

In this present study, we conducted a series of *in vivo* and *in vitro* experiments explore the antioxidative and neuroprotective effects of DJ-1 on HG-induced damage to RGCs. Our results suggest that DJ-1 represents a potential therapeutic target for early neuroprotection in DR.

## 2 Materials and methods

### 2.1 Animals

Wild-type (WT) C57BL/6J male mice were purchased from the Hunan SJA Laboratory Animal Co., Ltd. (Changsha, China), and DJ-1 deficiency (DJ-1^−/−^) male mice achieved by *PARK7* gene knockout (KO) were gifted by The State Key Laboratory of Medical Genetics of Central South University (Changsha, China). Mice were housed in the Central South University animal center individually in ventilated cages in a 14 h-light/10 h-dark cycle and were provided regular chow and water *ad libitum*. All procedures were approved and conducted in accordance with the Institutional Animal Care and Use Committee at the Second Xiangya Hospital, Central South University. Studies were conducted in accordance with the ARVO Statement Principles for the Use of Animals in Ophthalmic and Vision Research. Mice were genotyped before experiments.

Type 1 diabetes mellitus (T1DM) was induced in WT and DJ-1^−/−^ male mice through intraperitoneal injection of streptozotocin (STZ, 50 mg/kg, Sigma-Aldrich, St. Louis, United States) for five consecutive days (Liu et al., 2024; Li et al., 2020). A glucometer (Jiangsu Yuwell Medical Equipment and Supply Co., Ltd., Suzhou, China) was employed to monitor the level of blood glucose. Diabetic mice were used for subsequent experiments 4, 12, and 24 weeks after the final STZ injection, with the blood glucose level above 16.7 mmol/L, and the eyes were enucleated and processed for histological and molecular biology analyses. Four groups of mice, WT group, DJ-1^−/−^ group, WT T1DM group and DJ-1^−/−^ T1DM group, were used for animal experiments.

To verify the protective effect of DJ-1 on the retina, we overexpressed DJ-1 by intravitreal injection of adeno-associated viruses (AAV). The AAV of the negative control and DJ-1 overexpression vectors were constructed in the pAAV-CMV-MCS-EF1-mNeonGreen-WPRE vector by OBiO Technology (Shanghai) Co., Ltd. (Shanghai, China). The diabetic mice were randomly divided into two groups of 10 mice each: the DJ-1 overexpression (OE) group and the DJ-1 negative control (NC) group. We puncture at 1 mm posterior to the temporal limbus for each intraocular injection using a 30-gauge needle attached to a Hamilton syringe. One microliter of pAAV DJ-1 OE or NC plasmid was injected into the vitreous of each diabetic mouse in one eye. Eyes were enucleated and processed for histological and molecular biology analyses 12 weeks after injection.

### 2.2 R28 cell culture, infection, and treatment

Owing to the intricate process involved in the isolation, purification, and cultivation of primary RGCs, coupled with the low success rate, the expansion of primary cell culture poses a challenge. The R28 cell line is a rat retinal precursor cell line that exhibits biological similarities to RGCs. It is widely employed *in vitro* to investigate the neuroprotection, cytotoxicity, and physiological functions of RGCs (Seigel, 2014; Yao et al., 2023). R28 cells were cultured as previously described (Peng et al., 2022). In brief, cells were cultured in Dulbecco’s modified Eagle medium (DMEM, BasalMedia, Shanghai, China) containing 10% fetal bovine serum (FBS, Gibco, Waltham, United States) and 1% antibiotic-antimycotic (Gibco, Waltham, United States) at 37°C with 5% CO_2_. The logarithmic growth phase cells fed with a medium containing either HG (30 mM D-glucose) or normal glucose (NG, 5.6 mM D-glucose) for 24 h were used for the following experiments.

The lentiviruses of DJ-1 OE and DJ-1 NC vectors were constructed in the pcSLenti-EF1-EGFP-P2A-Puro-CMV-MCS-3xFLAG-WPRE lentiviral vector by OBiO Technology (Shanghai) Co., Ltd. (Shanghai, China). Briefly, R28 cells were seeded in 12-well culture plates and infected with vectors at 40% confluence. The medium was changed after 12 h. After 72 h, the EGFP fluorescence was observed under a fluorescence microscope (Leica, Wetzlar, Germany) to determine the efficiency of the lentivirus infection. Positive clones were screened with puromycin (Sigma, St. Louis, United States), and the expression of DJ-1 was detected by Western blotting to determine whether stable cell lines were successfully constructed.

### 2.3 Hematoxylin and eosin (HE) staining

Hematoxylin and eosin (HE) staining was used to observe the histological structure of retinal tissue. Briefly, 4% paraformaldehyde-fixed and paraffin-embedded eyeballs were cut into 4 µm thickness along the vertical meridian of the optic disc in each eye. After dewaxing with dimethylbenzene and dehydrating with graded ethanol series, the slices were stained with hematoxylin for 5 min and then eosin for 30 s. The tissues were visualized using a microscope (Axio Imager M2, Carl Zeiss Microscopy GmbH, Germany) following sealing with neutral gum.

### 2.4 Immunofluorescence staining

Fresh mouse eyeball tissue was extracted without fixation and directly embedded in OCT for frozen sectioning. Frozen sections (10 µm) were fixed by acetone, permeabilized by 1% Triton X-100, and blocked with 10% normal goat serum (Beyotime, Shanghai, China). The sections were then incubated with the rabbit anti-DJ-1 antibody in PBS with 5% BSA overnight at 4°C. Bound antibodies were detected with DyLight 488-conjugated IgG in PBS with 5% BSA. The detailed information on the antibodies is provided in Supplementary Table S1. A fluorescence microscope (Axio Imager M2, Carl Zeiss Microscopy GmbH, Germany) was used for imaging after incubating with DAPI (Genview Inc., Shanghai, China) for nuclear counterstaining and mounting.

### 2.5 Whole-mount retina immunolabeling and RGC counting

RGCs in whole-mounted retinas were assessed by observing flat-mounted retinas. The eyes of mice were enucleated and fixed in 4% PFA for 2 h at room temperature after the cornea and lens were removed. The retinas were separated and fixed in 4% PFA overnight. The fixed retinas were transferred to a glass slide and cut into four equal quadrants. Retinas were permeabilized and blocked in PBS containing 0.5% Triton X-100% and 1% BSA for 1 h at room temperature. RGCs were labeled with anti-β III Tubulin overnight at 4°C, and detected by DyLight 488-conjugated IgG for 1 h at room temperature, followed by mounting. The detailed information on the antibodies is provided in Supplementary Table S1. Images were captured using a fluorescence microscope (Axio Imager M2, Carl Zeiss Microscopy GmbH, Germany). Each retina was divided into four quadrants (sharing a central quadrant), and β III Tubulin-positive cells were counted using ImageJ software.

### 2.6 Electroretinogram (ERG)

Flash electroretinographic recordings were carried out following the International Society for Clinical Electrophysiology of Vision (ISCEV) recommendations. Briefly, after a 12 h-dark-adaption, mice were anesthetized with an intraperitoneal injection of ketamine (80 mg/kg, Henry Schein, NY, United States) and xylazine (16 mg/kg, Sigma-Aldrich, St. Louis, United States). Topical anesthesia was obtained by obuvacaine hydrochloride eye drops (Santen Pharmaceutical Co., Osaka, Japan), and pupils were dilated with 0.5% tropicamide (Akorn, Inc., Lake Forest, United States). A reference electrode was placed subcutaneously at the base, and the electrode placed on the tail was the ground electrode. Retinal responses were recorded simultaneously from both eyes with bipolar electrodes placed on the corneas. We recorded dark adaptation intensity 0.001–3.0 cd s/m^2^ white flash stimulus and dark adaptation 3.0 oscillatory potential by RetiMINER visual electrophysiology device (Erxi Co. LTD., Chongqing, China). The ERG and waveform were analyzed. Baseline values are the mean of the signal at the first 19.5 ms before stimulation. The amplitude of the a-wave was measured between the baseline and the local minimum of the signal, and the amplitude of the b-wave was measured between the a-trough and the local maximum of the signal. The amplitude of oscillatory potentials was recorded at the intensity of 3.0 cd s/m^2^, which was determined to be the sum of three major amplitudes.

### 2.7 Apoptosis assay

Apoptosis was evaluated by Terminal deoxynucleotidyl transferase dUTP nick end labeling (TUNEL) staining, which was performed using a TUNEL Bright Red Apoptosis Detection kit (Vazyme, Nanjing, China), following the manufacturer’s instructions. Briefly, cells on coverslips were fixed by paraformaldehyde and permeabilized by Triton X-100. The prepared cells were labeled with TdT reaction mix for 1 h at 37°C and incubated with DAPI (Genview Inc., Shanghai, China) for nuclear counterstaining. After mounting the coverslips on slices, images were captured on a fluorescence microscope (Axio Imager M2, Carl Zeiss Microscopy GmbH, Germany).

### 2.8 ROS measurement

The ROS measurement was performed with the Reactive Oxygen Species Assay Kit (Beyotime, Shanghai, China). In short, the cells were incubated 30 min in the dark at 37°C with DCFH-DA (1:1,000) and washed thrice with serum-free medium. Fluorescent cells were displayed using a fluorescence microscope (Leica, Wetzlar, Germany) with an excitation wavelength of 488 nm.

### 2.9 Mitochondrial membrane potential (ΔΨm) detection

The *ΔΨm* was detected by the JC-1 detection kit (Solarbio, Beijing, China) according to the instructions. R28 cells were washed and incubated with JC-1 working solutions for 30 min at 37°C in the incubator. After incubation, live cells were imaged on a fluorescence microscope (Leica, Wetzlar, Germany). JC-1 forms JC-1 aggregates exhibiting red fluorescence at high *ΔΨm*, whereas the JC-1 monomer shows green fluorescence at a low *ΔΨm*. The *ΔΨm* of healthy mitochondria is typically maintained at a high level. Changes in the fluorescence ratio of the red to green fluorescence were used to assess mitochondrial function status.

### 2.10 Total protein extraction and Western blot (WB) analysis

Briefly, cells and retinas were homogenized in radioimmunoprecipitation assay (RIPA) lysis buffer (Service, Wuhan, China) containing protease inhibitor cocktail (APExBio, Houston, United States) and PMSF (Solarbio, Beijing, China). The samples were fully lysed by sonication on ice and centrifuged at 12,000 g for 10 min at 4°C. The supernatant was collected and quantitated with a BCA protein quantification kit (Boster, Wuhan, China).

Protein extracts were separated on 12% SDS–polyacrylamide gel electrophoresis (SDS-PAGE) and blotted onto PVDF membranes (Millipore Sigma, St. Louis, United States). Membranes were blocked in 5% fat-free milk (Biosharp, Beijing, China) at room temperature for 1 h, and then exposed to primary and secondary antibodies. The detailed information on the antibodies is provided in Supplementary Table S1. The blots were scanned with the ChemiDoc MP System (Bio-Rad, Hercules, United States) and processed using ImageJ software. For quantification, proteins were normalized to β-actin.

### 2.11 Statistical analyses

Data were analyzed using GraphPad Prism (v9.0.1 for macOS) software (GraphPad Software, San Diego, CA, United States) and are presented as the mean ± standard deviation (SD). Unpaired, two-tailed t-test and one-way or two-way ANOVA with Tukey’s Multiple Comparisons test were used for determining statistical significance between groups. *P* values less than 0.05 were statistically significant.

## 3 Results

### 3.1 Deletion of DJ-1 exacerbates structural and functional impairment in diabetic neuroretina

An STZ-induced mouse model was utilized, demonstrating consistent characteristics of T1DM in terms of body weight and blood glucose levels (Supplementary Figure S1). This model was employed to explore the influence of elevated glucose levels on retinal structure, function, and protein expression.

**FIGURE 1:**
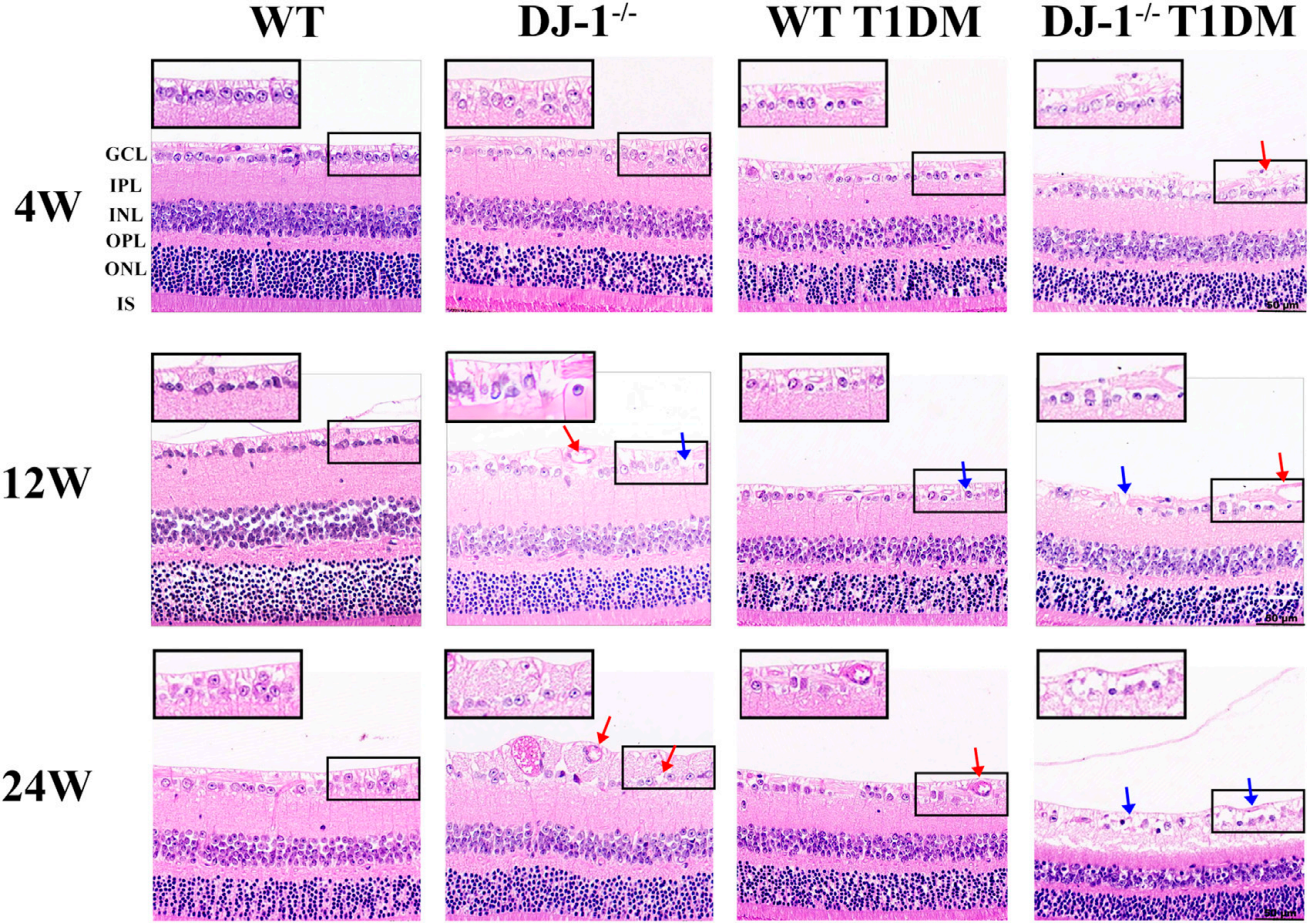
DJ-1 knockout exacerbates retinal structure impairment in diabetic mice. In wild-type or DJ-1^−/−^ mice with or without diabetes 4, 12, and 24 weeks, the retinal structure was assessed by hematoxylin-eosin staining (n = 6 per group). Red arrows represent vacuolar changes, and blue arrows represent structural breaks or discontinuities. The scale bar represents 50 μm, with an enlarged inset highlighting the boxed area. GCL: ganglion cell layer; IPL: inner plexiform layer; INL: inner nuclear layer; OPL: outer plexiform layer; ONL: outer nuclear layer; IS: inner segments of photoreceptors.

HE staining was conducted on paraffin-embedded retinal sections to provide a comprehensive assessment of structural alterations within the retina. Notably, GCL exhibited disordered structure and vacuole-like changes in DJ-1^−/−^ mice and both diabetic groups when compared to the WT control group (Figure 1). At 24 weeks, DJ-1^−/−^ T1DM mice displayed the most pronounced structural damage in the GCL, characterized by rupture and separation. These findings collectively underscore the detrimental impact of DJ-1 deficiency and diabetes on retinal structural integrity, with a particular emphasis on the exacerbating effect when these factors coexist.

**FIGURE 2:**
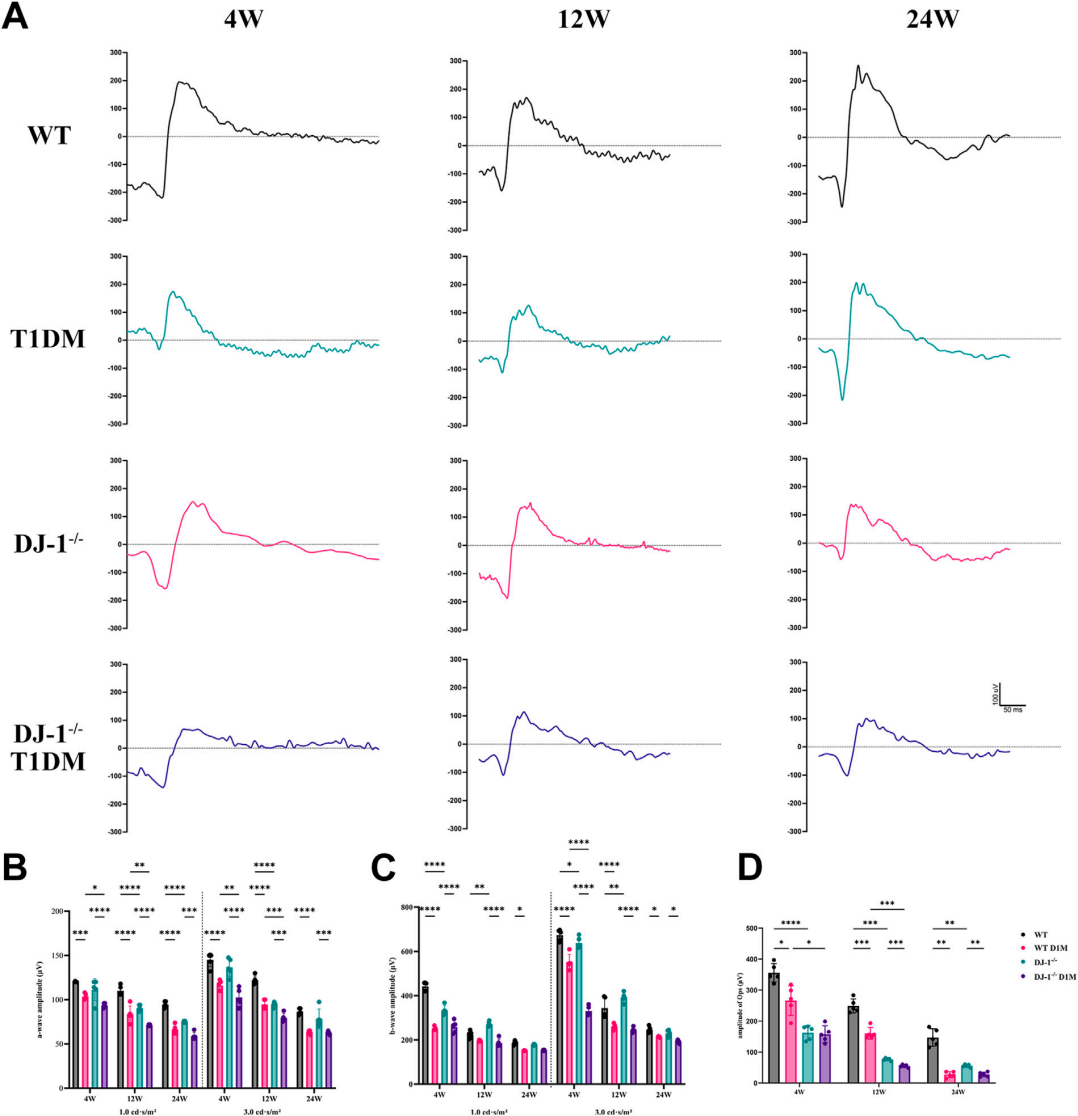
DJ-1 knockout exacerbates retinal function impairment in diabetic mice. **(A)** Representative profile of ERG for wild-type and DJ-1^−/−^ mice with or without diabetes; **(B–D)** Quantitative analysis of a-wave, b-wave, and OPs, respectively (n = 5 per group). OPs: oscillatory potentials. The comparison was based on two-way ANOVA analysis. *P* values of <0.05, <0.01, <0.001, and <0.0001 are indicated by *, **, ***, and ****, respectively.

ERG serves as a vital tool for assessing retinal integrity and function. The amplitudes of both a-wave and b-wave exhibited an increase with the intensity of flash stimulation, reaching significant differences at a light intensity of 1.0 cd s/m^2^ (Supplementary Figure S2). Upon curve fitting the waveform at the light intensity of 1.0 cd s/m^2^, the amplitudes of the b-wave demonstrated a decrease in both DJ-1^−/−^ and T1DM groups compared to the WT group at 4, 12, and 24 weeks (Figure 2A). To comprehensively understand retinal function alterations, statistical analyses were performed on the amplitudes of the a-wave and b-wave at light intensities of 1.0 cd s/m^2^ and 3.0 cd s/m^2^. Both the a-wave and b-wave amplitudes were statistically significantly reduced in both DJ-1^−/−^ and T1DM groups compared to the WT group at 4, 12, and 24 weeks, with DJ-1^−/−^ T1DM mice experiencing the most severe decrease (Figures 2B, C). Oscillatory potentials (OPs) exhibited decreased amplitudes in both DJ-1^−/−^ and T1DM groups compared to the WT group (Figure 2D).

**FIGURE 3:**
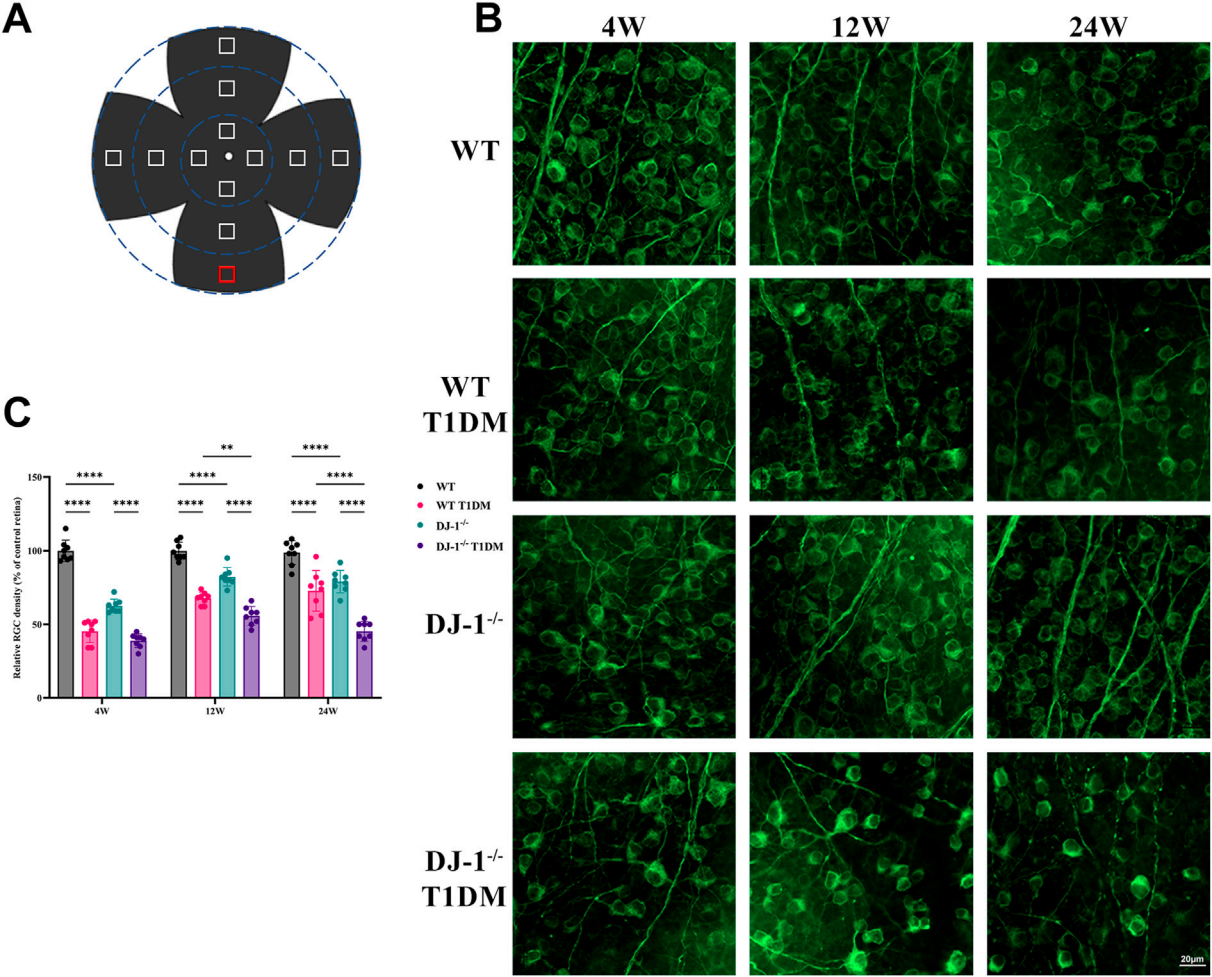
DJ-1 knockout exacerbates RGC loss in diabetic mice. **(A)** A schematic diagram of retina whole mounting; **(B)** In wild-type or DJ-1^−/−^ mice with or without diabetes 4, 12, and 24 weeks, β III Tubulin-positive cells in whole mounted retinas were assessed by observing flat-mounted retinas, scale bar 20 μm; **(C)** Quantitative analysis of RGC number (n = 8 per group). The comparison was based on two-way ANOVA analysis. *P* values of <0.01, and <0.0001 are indicated by **, and ****, respectively.

RGCs play a crucial role in neural function and visual quality. The β III Tubulin staining was conducted on retinal flat mounts to assess the survival of RGCs. Each flat-mounted retina was divided into four quadrants sharing a central quadrant and divided into three zones from 1/6, 1/2, and 5/6 radii from the optic disc in each quadrant (Figure 3A). The β III Tubulin-positive cells in each zone were manually counted to calculate the mean values for RGC density (i.e., RGCs per square millimeter) determination. The relative RGC density of each group was calculated as a percentage of the mean value, with the WT retina as a reference. The staining revealed a progressive decline in the number of RGCs with duration of diabetes. This process was accelerated by DJ-1^−/−^. DJ-1^−/−^ T1DM mice exhibited the most severe RGC loss from 4 weeks to 24 weeks (Figures 3B, C). As anticipated, these findings underscored that the absence of DJ-1 heightened the loss of RGCs in diabetic mice.

**FIGURE 4:**
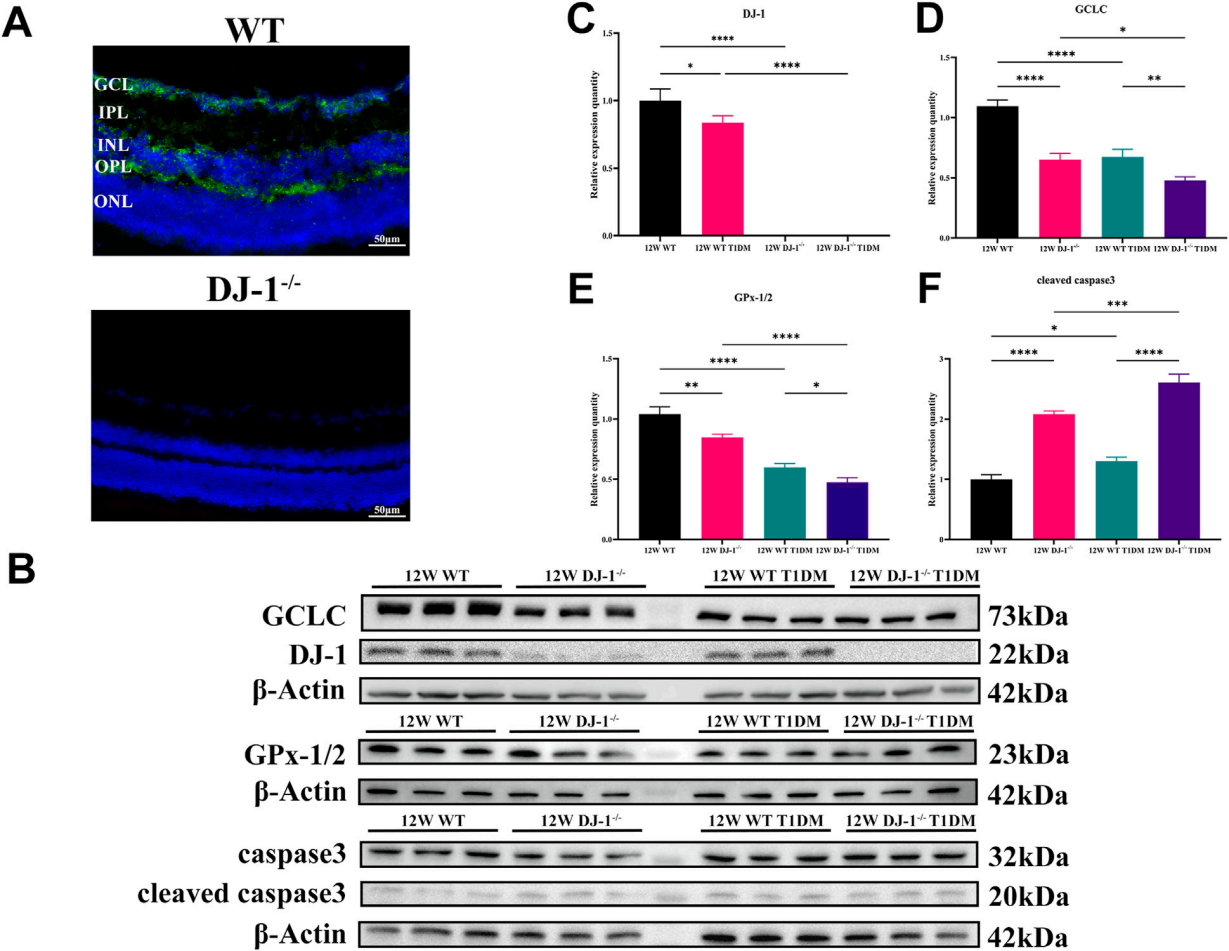
DJ-1 knockout exacerbates abnormalities in retinal protein expression. **(A)** Immunofluorescence staining showed the distribution of DJ-1 protein in the retina, scale bar 50 μm; **(B)** Western blotting of 12-week wild-type or DJ-1^−/−^ mice with or without diabetes, including DJ-1, GCLC, caspase3, and GPx-1/2 (n = 3 per group); **(C–F)** Quantitative analysis of Western blot results for DJ-1, GCLC, caspase3, and GPx-1/2, respectively. GCL: ganglion cell layer; IPL: inner plexiform layer; INL: inner nuclear layer; OPL: outer plexiform layer; ONL: outer nuclear layer. The comparison was based on one-way ANOVA analysis. *P* values of <0.05, <0.01, <0.001, and <0.0001 are indicated by *, **, ***, and ****, respectively.

Additionally, the localization of DJ-1 in the retina was examined via immunofluorescence. The staining revealed widespread distribution of DJ-1 throughout retinal layers, including the GCL (Figure 4A). Conversely, no positive signal was observed in the DJ-1^−/−^ sample.

Protein expression analysis at 12 weeks demonstrated the expression of antioxidant proteins DJ-1, GCLC, and GPx-1/2 in the retina of WT T1DM mice was significantly downregulated compared to the WT group (Figures 4B–E). DJ-1^−/−^ also significantly inhibited the expression of GCLC and GPx-1/2 compared to the WT group. Meanwhile, the expression of the antioxidant protein decreased most significantly in the DJ-1^−/−^ T1DM group. (Figures 4D, E). Furthermore, cleaved caspase3 was statistically elevated in both DJ-1^−/−^ and DJ-1^−/−^ diabetic retinas (Figure 4F). These results collectively indicated that the STZ-induced model exhibited typical diabetic retinal changes accompanied by redox system disorders. The knockout of DJ-1 contributed to retinal structural and functional disorders, exacerbating the dysregulated expression of oxidation-related proteins in the diabetic retina.

### 3.2 Overexpression of DJ-1 mitigated structural and functional impairment in diabetic neuroretina

We locally overexpressed DJ-1 in the retina through intravitreal injection of AAV to investigate its protective role against HG injury *in vivo*. The intravitreal AAV injection effectively led to retinal infection (Supplementary Figure S3A), resulting in significantly higher DJ-1 expression in the retina of the OE group compared to the control and NC groups (Supplementary Figure S3B, C).

**FIGURE 5:**
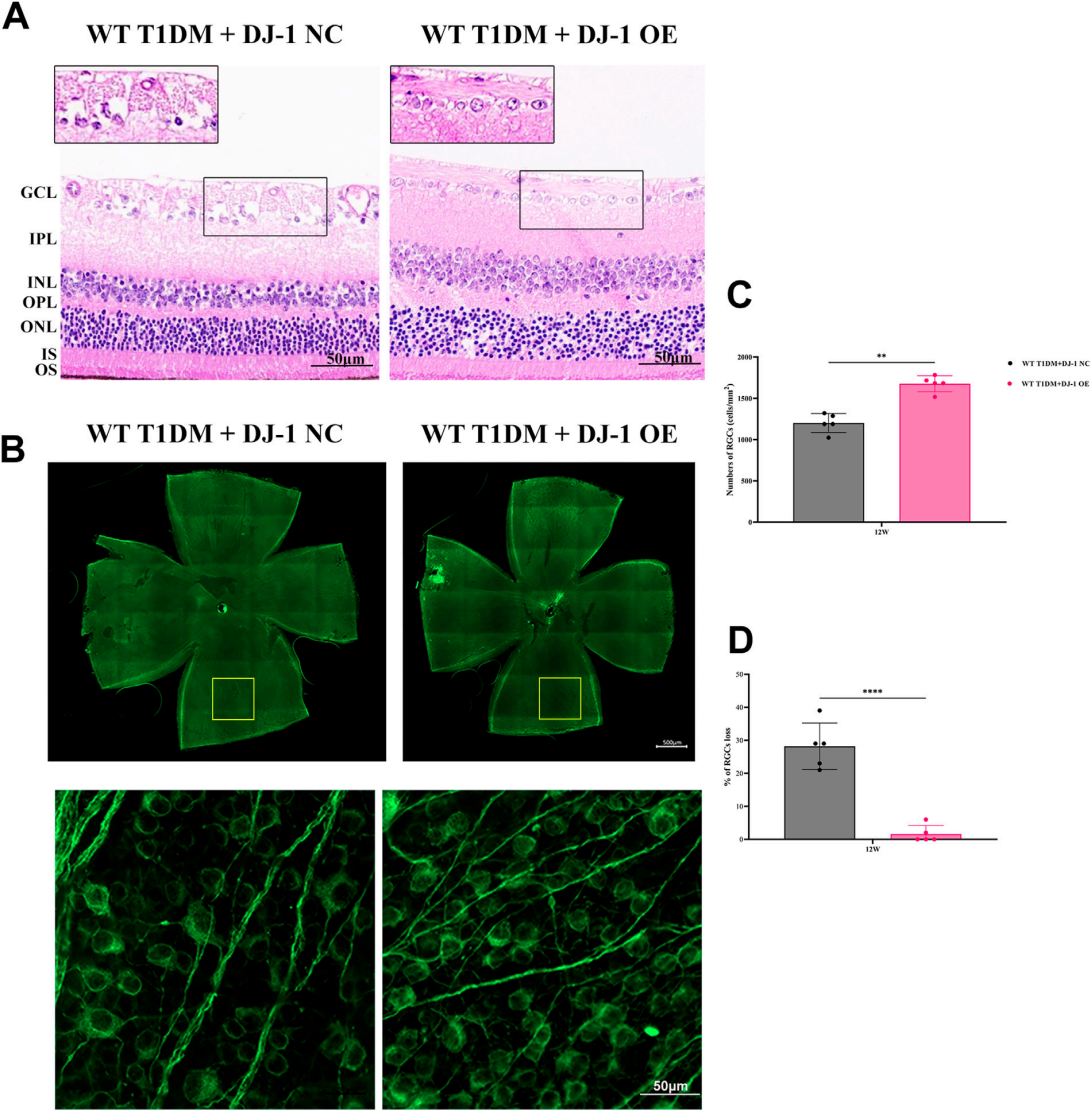
DJ-1 overexpression rescues diabetic abnormalities in the retinal structure and RGC apoptosis. **(A)** Hematoxylin-eosin staining showed retinal structure in mice with intravitreal injection of *PARK7* overexpression virus and control virus (n = 5 per group). The scale bar represents 50 μm, with an enlarged inset highlighting the boxed area; **(B)** The β III Tubulin-positive cells in whole mounted retinas were assessed by observing flat-mounted retinas (n = 5 per group), scale bar 500 μm, the picture below is an enlargement of the one above, scale bar 50 μm; **(C–D)** Quantitative analysis of RGC number. RGC: retinal ganglion cell; GCL: ganglion cell layer; IPL: inner plexiform layer; INL: inner nuclear layer; OPL: outer plexiform layer; ONL: outer nuclear layer; IS/OS: inner segments of photoreceptors/outer segments of photoreceptors; NC: negative control; OE: overexpression. The comparison was based on an unpaired two-tailed t-test. *P* values of <0.01, and <0.0001 are indicated by ** and ****, respectively.

Histological evaluation through HE staining revealed a vacuole-like change and disordered structure of the GCL in the DJ-1 NC group compared to the DJ-1 OE group (Figure 5A). Additionally, the observation of whole-mounted retinas stained with β III Tubulin demonstrated that DJ-1 OE significantly alleviated diabetes-induced RGC death, whereas the NC virus had no effect (Figures 5B–D).

**FIGURE 6:**
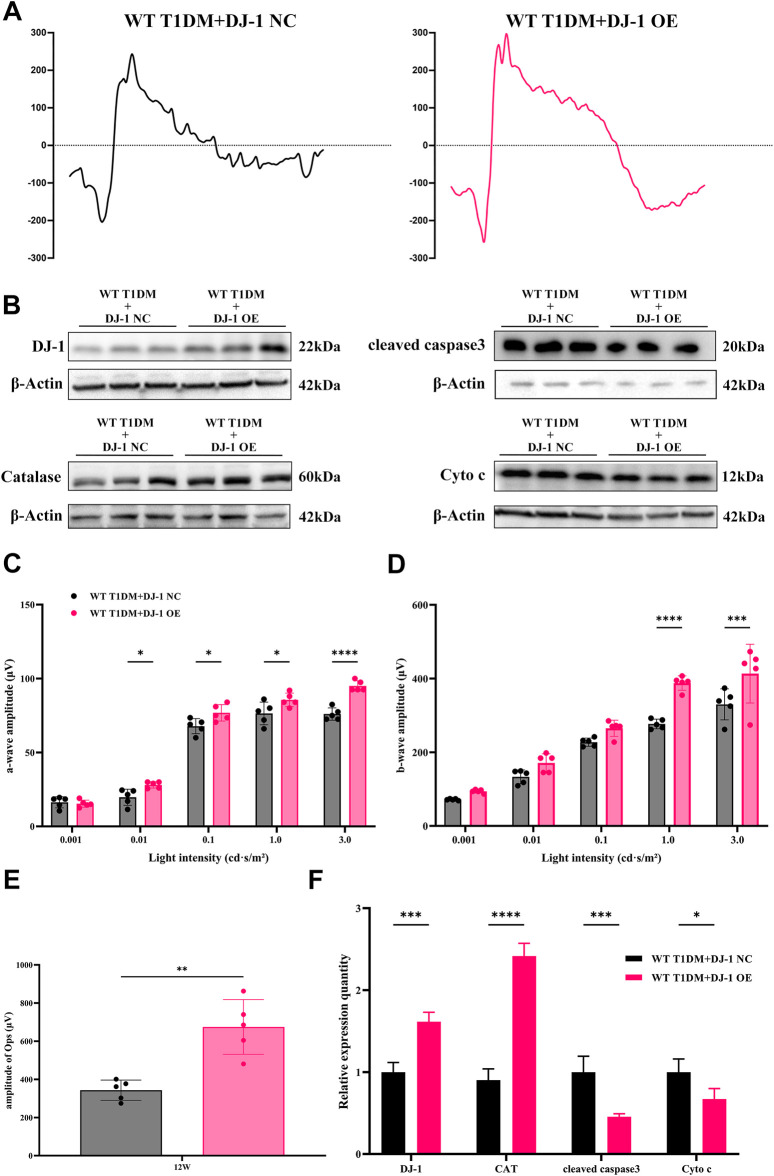
DJ-1 overexpression rescues diabetic retinal function and oxidative stress. **(A)** Representative profile of ERG for diabetic mice with DJ-1 NC and OE virus (n = 5 per group); **(B)** Western blotting was used to detect protein expression in diabetic mice with DJ-1 NC and OE virus, including DJ-1, Catalase, caspase3, and Cyto c (n = 3 per group); **(C–E)** Quantitative analysis of a-wave, b-wave, and OPs, respectively; **(F)** Quantitative analysis of Western blot results for DJ-1, Catalase, caspase3, and Cyto c. NC: negative control; OE: overexpression. The comparison was based on an unpaired two-tailed t-test. *P* values of <0.05, <0.01, <0.001, and <0.0001 are indicated by *, **, ***, and ****, respectively.

ERG analysis (Figures 6A, C–E) revealed that DJ-1 OE significantly mitigated the diabetes-induced reduction in the amplitudes of the a-wave, b-wave, and OPs. To further investigate redox protein expression, WB analysis was performed on retinas pretreated with DJ-1 NC and OE vectors. The results demonstrated that DJ-1 OE led to significant alterations in protein expression levels, notably increasing DJ-1 and Catalase expression while suppressing cleaved caspase3 and Cyto c expression (Figures 6B, F). These findings underscore the potential of DJ-1 as a therapeutic target for protecting against diabetes-induced retinal damage by modulating redox homeostasis.

### 3.3 HG induces oxidative stress, mitochondrial dysfunction and apoptosis in R28 cells

**FIGURE 7:**
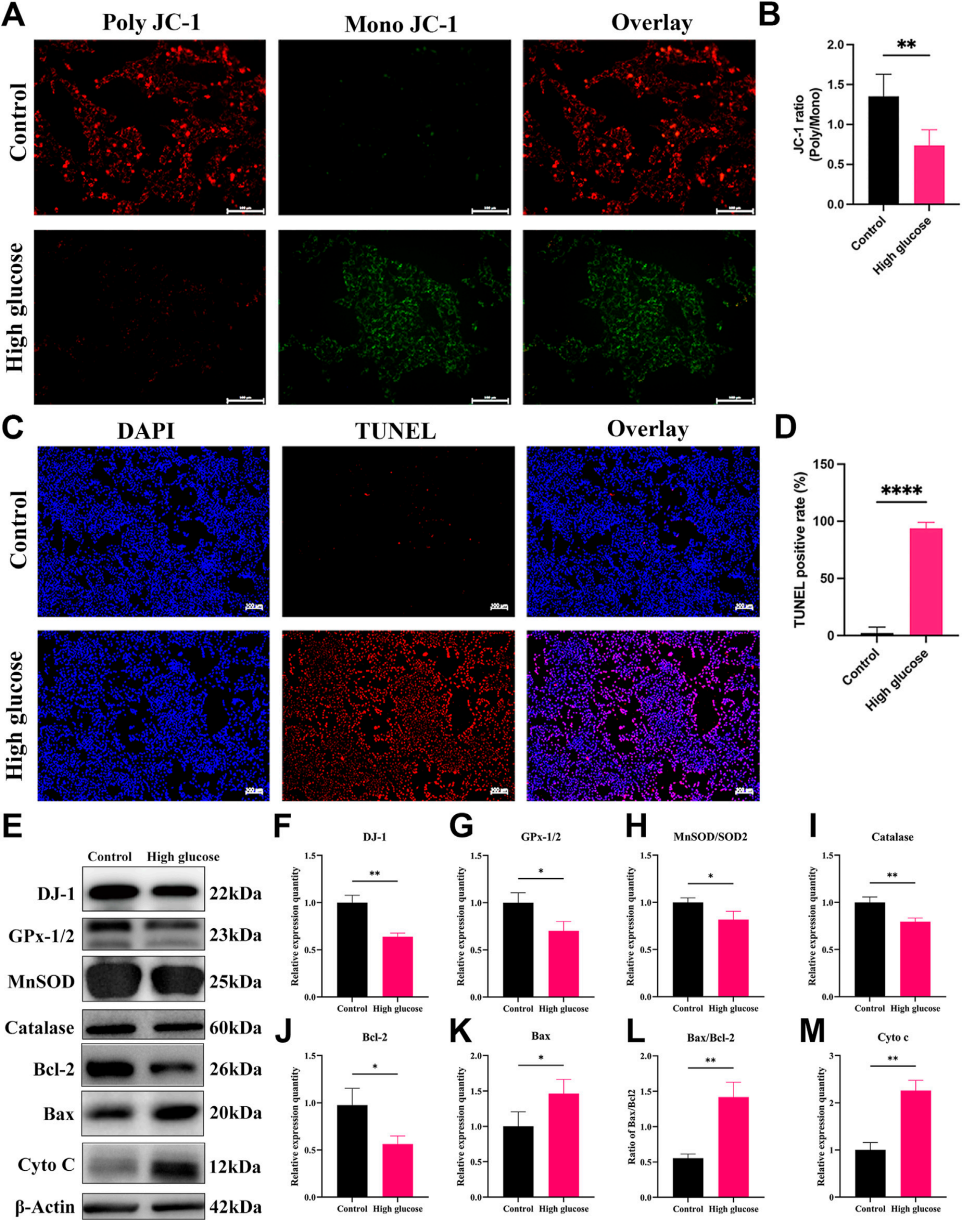
High glucose induces mitochondrial damage, apoptosis, and oxidative stress in R28 cells. **(A)** JC-1 staining showed mitochondrial transmembrane potential in R28 cells in normal control and high glucose treatment (n = 3 independent experiments), scale bar 100 μm; **(B)** Quantitative fluorescence density results of JC-1 ratio (poly/mono); **(C)** TUNEL staining showed R28 cell apoptosis (n = 3 independent experiments), scale bar 100 μm; **(D)** Quantitative analysis of apoptotic cell number results. **(E)** Western blotting results showed that high glucose challenge induced changes in DJ-1, GPx-1/2, MnSOD/SOD2, Catalase, Bcl-2, Bax, Bax/Bcl-2, and Cyto c protein expression (n = 3 independent experiments); **(F–M)** Quantitative analysis of Western blot results for DJ-1, GPx-1/2, MnSOD/SOD2, Catalase, Bcl-2, Bax, Bax/Bcl-2, and Cyto c, respectively. TUNEL: Terminal deoxynucleotidyl transferase dUTP nick end labeling. The comparison was based on an unpaired two-tailed t-test. *P* values of <0.05, <0.01, and <0.0001 are indicated by *, **, and ****, respectively.

To decipher the mechanisms underlying HG-induced impairment in DR, we conducted an investigation into the effects of HG on cultured R28 cells. The *ΔΨm* is pivotal in determining mitochondrial bioenergetics and cell fate under oxidative stress. A decline in *ΔΨm* typically occurs in the early stages of apoptosis. We employed JC-1 staining to assess changes in *ΔΨm*, revealing a substantial decrease in the JC-1 ratio (poly/mono) in the HG group (Figures 7A, B), indicating a significant reduction in *ΔΨm* in R28 cells under HG conditions. To investigate whether HG-induced RGC death occurs through an apoptotic mechanism, we quantified apoptotic R28 cells using chromatin staining and TUNEL in both HG and NG cultures. Staining results demonstrated a marked increase in TUNEL-positive cell numbers in R28 cells under HG compared to the NG group (Figures 7C, D).

**FIGURE 8:**
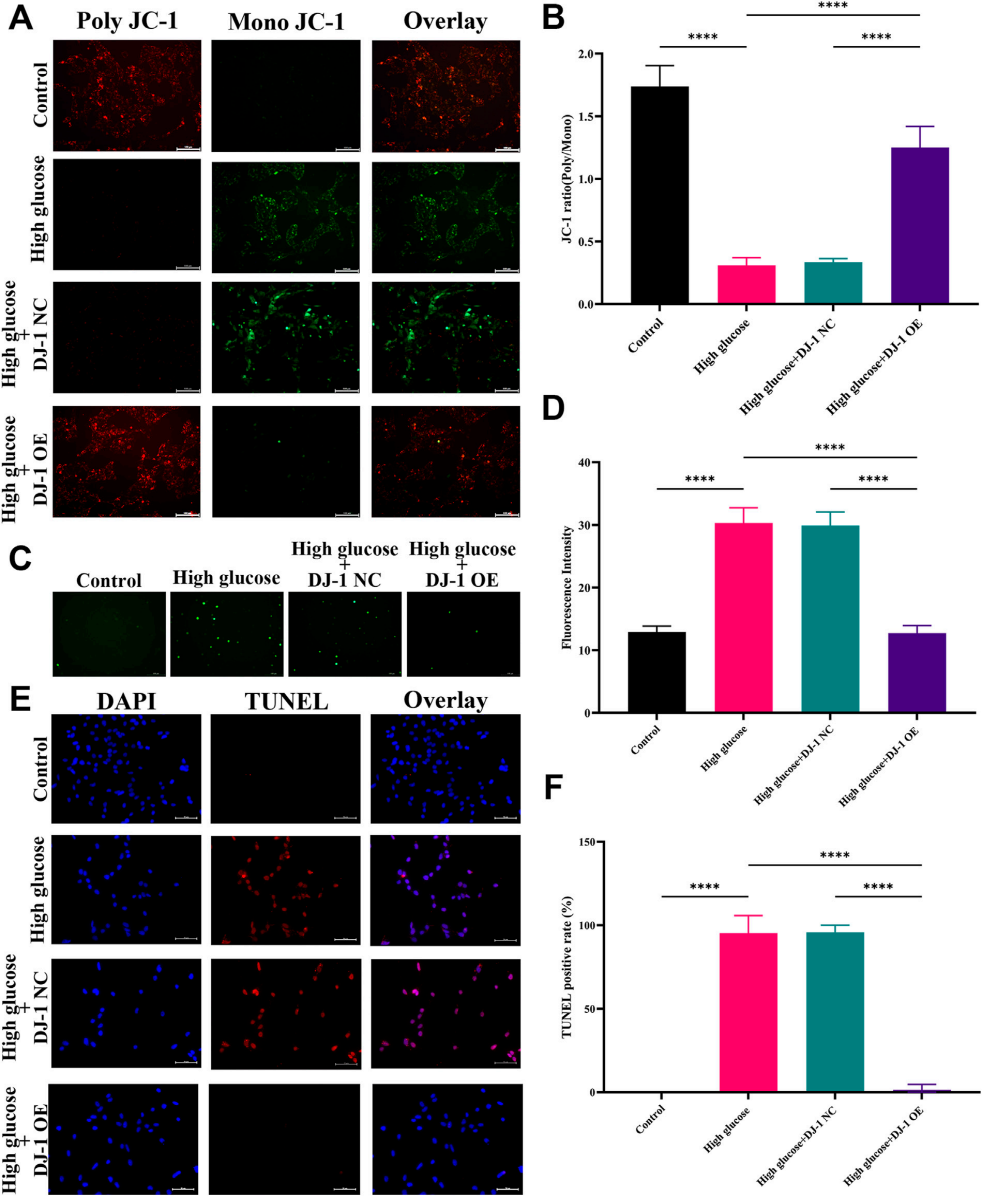
DJ-1 regulates mitochondrial function, ROS generation, and cell apoptosis in R28 cells. **(A)** JC-1 staining showed mitochondrial transmembrane potential in R28 cells in control, high glucose, and high glucose with lentiviruses treatment (n = 3 independent experiments), scale bar 100 μm; **(B)** Quantitative fluorescence density results of JC-1 ratio (poly/mono); **(C)** ROS staining showed ROS generation in R28 cells in control, high glucose, and high glucose with lentiviruses treatment (n = 3 independent experiments); **(D)** Quantitative fluorescence density results of ROS generation; **(E)** TUNEL staining showed that DJ-1 overexpression significantly decreased the number of apoptotic R28 cells induced by high glucose (n = 3 independent experiments), scale bar 50 μm; **(F)** Quantitative analysis of apoptotic cell number results. ROS: reactive oxygen species; TUNEL: Terminal deoxynucleotidyl transferase dUTP nick end labeling; NC: negative control; OE: overexpression. The comparison was based on one-way ANOVA analysis. *P* values of <0.0001 are indicated by ****.

Considering that ROS is a primary contributor to diabetic conditions, we observed a significant increase in ROS production in R28 cells exposed to HG (Figure 8C). The regulation of oxidative stress is contingent on the expression of antioxidant enzymes critical for mitochondrial protection against oxidative stress. WB analysis revealed a significant decrease in the expression of antioxidant and antiapoptotic proteins, including DJ-1, Bcl-2, GPx-1/2, MnSOD, and Catalase in the HG group compared to the NG group (Figures 7E–J). Meanwhile, Bax expression, the ratio of Bax to Bcl-2, and Cyto c expression significantly increased (Figures 7K, L). The relative expression quantity of Cyto c, a marker of mitochondrial dysfunction, significantly increased (Figure 7M). These results indicate that HG-induced damage involves mitochondrial dysfunction and apoptosis, possibly attributed to redox cofactor imbalance and heightened ROS production.

### 3.4 Overexpression of DJ-1 attenuated oxidative stress, mitochondrial dysfunction and apoptosis in R28 cells

We aimed to ascertain whether DJ-1 could safeguard RGCs against HG-induced oxidative stress by employing lentiviruses to induce DJ-1 overexpression in R28 cells. Four multiplicities of lentivirus infection (MOI) were tested: MOI 10, MOI 20, MOI 40, and MOI 80. Fluorescence analysis revealed that MOI 40 was the optimal infection condition (Supplementary Figure S4A). Correspondingly, at MOI 40, the DJ-1 expression level in the OE group significantly surpassed that in the NC group and control group, while no significant difference was observed between NC and the control group (Supplementary Figures S4B, C). Additionally, we evaluated cellular *ΔΨm*, ROS production, and apoptosis under NG, HG, and virus-infected HG conditions.

Consistent with our prior findings, JC-1 staining demonstrated a significant reduction in the JC-1 ratio (poly/mono) under HG, indicative of reduced *ΔΨm*. Notably, DJ-1 overexpression significantly mitigated HG-induced *ΔΨm* loss, whereas the NC group exhibited no such effect (Figures 8A, B). Similarly, DJ-1 overexpression markedly decreased HG-induced ROS production, whereas the NC vector had no impact (Figures 8C, D). As anticipated, DJ-1 overexpression significantly attenuated TUNEL staining-positive cells caused by HG compared to the NG group (Figures 8E, F).

**FIGURE 9:**
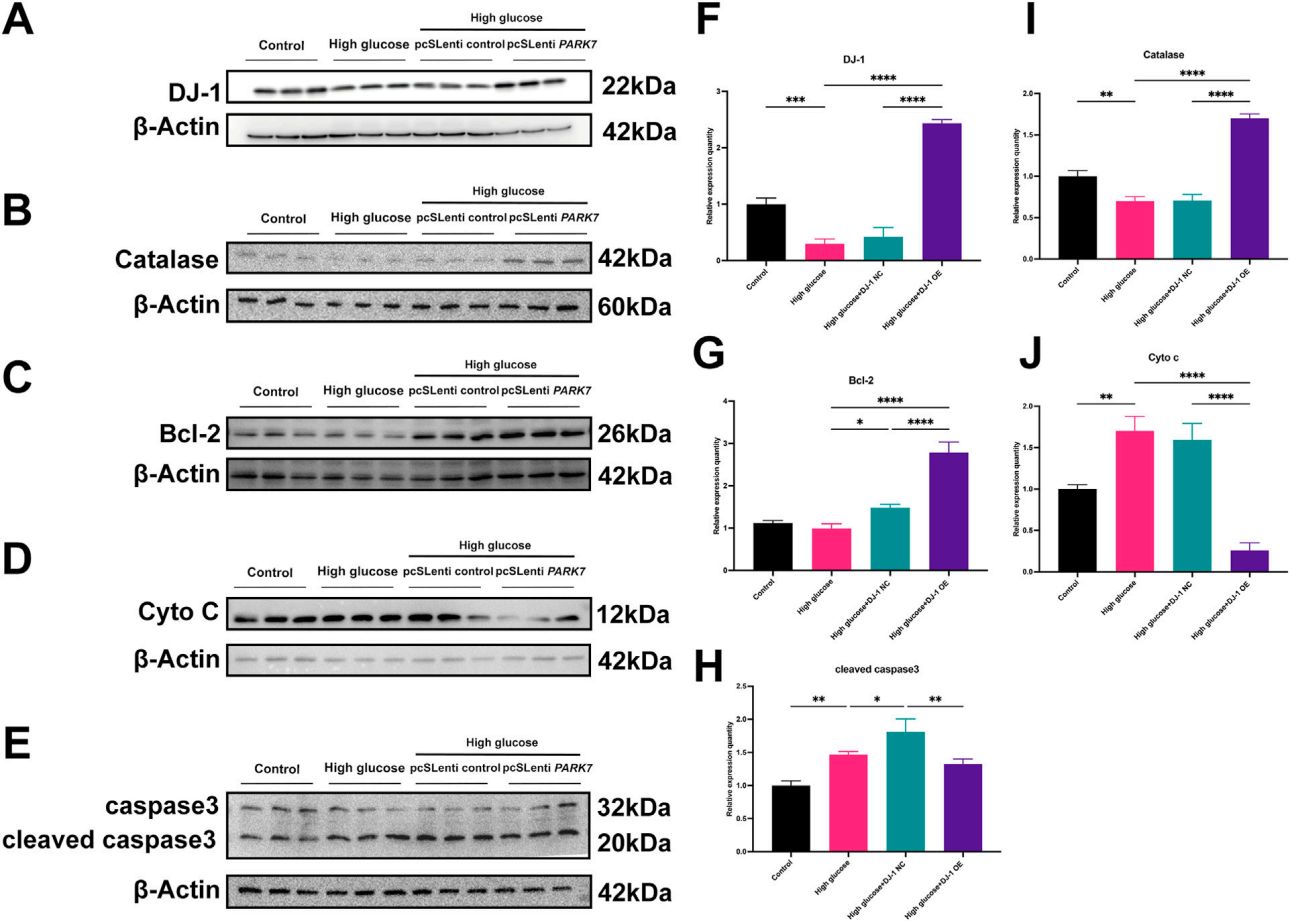
DJ-1 overexpression rescues HG-induced disturbances in redox and apoptotic protein expression. **(A–E)** Western blotting results showed that DJ-1 overexpression significantly reversed high glucose-induced protein expression abnormalities, including DJ-1, Catalase, Bcl-2, Cyto c, and caspase3 (n = 3 independent experiments); **(F–J)** Quantitative analysis of Western blot results for DJ-1, Catalase, Bcl-2, Cyto c, and caspase3, respectively. The comparison was based on one-way ANOVA analysis. *P* values of <0.05, <0.01, <0.001, and <0.0001 are indicated by *, **, ***, and ****, respectively.

Our previous investigations revealed that HG diminished the expression levels of antioxidant proteins while increasing pro-oxidative and proapoptotic protein expressions. DJ-1, a mitochondrial protection and antioxidant protein, combats HG damage in various retinal cells. In R28 cells, DJ-1 overexpression significantly elevated Bcl-2 and Catalase expression levels while inhibiting Cyto c expression (Figure 9). Although the results did not reach statistical significance, overexpression of DJ-1 under high glucose conditions visibly inhibited the expression of cleaved caspase3. These results demonstrated that HG induces redox imbalance and cell death in R28 cells, and DJ-1 overexpression significantly alleviates this injury, highlighting the essential role of DJ-1 in protecting RGCs from HG-induced damage.

## 4 Discussion

**FIGURE 10:**
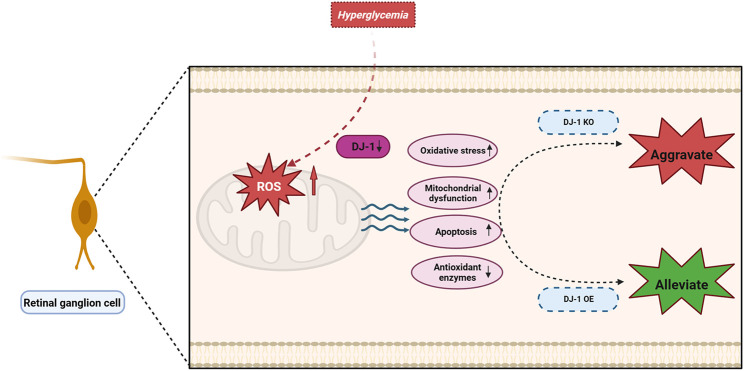
DJ-1 enhances mitochondrial function and reduces oxidative stress, thereby promoting retinal ganglion cell survival in diabetic retinopathy.

In this investigation, we elucidated that the detrimental impact on RGCs induced by a HG environment primarily stems from mitochondrial dysfunction and oxidative stress. Furthermore, our findings emphasized the protective role of DJ-1 in mitigating ROS elevation in the HG-induced R28 model, thereby shielding the diabetic retina from oxidative damage, mitochondrial dysfunction, and apoptosis. These outcomes propose DJ-1 as a prospective antioxidant for DR, acting through the regulation of mitochondrial function and oxidative stress (Figure 10).

The pathogenesis of DR is intricate, with unclear underlying mechanisms. Retinal neurodegeneration, preceding microvascular changes, emerges as an initial event contributing to DRV, detectable before clinical manifestation (Sohn et al., 2016; Carrasco et al., 2007; Amato et al., 2022). Neurodegenerative alterations, characterized by excitotoxicity, increased neuronal apoptosis, glial cell reactivity, microglial activation, glutamate metabolism shifts, and neurotrophin depletion, typify the early stages of DR under hyperglycemic conditions (Hernandez et al., 2016). Neuronal apoptosis, a hallmark of neurodegenerative diseases (Kadlubowska et al., 2016; Gorman, 2008), encompasses various types of neuronal death, including RGCs, under hyperglycemic conditions (Lam et al., 2023; Pal et al., 2020). RGC apoptosis significantly contributes to the onset and mediation of early DR (Lim et al., 2018; Rolev et al., 2021).

RGCs, pivotal for transmitting retinal signals to the central nervous system (Kim et al., 2021), face heightened vulnerability to metabolic stressors and ROS, especially in diabetes (Ito and Di Polo, 2017). The mode and mechanism of RGC death may vary in different lesions (e.g., mechanical injury, inflammation, hypoxia, ischemia, oxidative stress, and hyperglycemia). More specifically, the molecular mechanisms of somal and axonal degeneration are distinct, and the latter can result in retrograde somal degeneration (Zuo et al., 2023). Due to RGCs’ trajectory and space constraints coursing through the optic nerve and retrograde involvement, diabetic RGC loss will gradually become apparent. Unlike cell lines, adult mammalian RGCs cannot proliferate or regrow, and their axons have little capacity to regenerate following damage. In other words, RGC loss equals irreversible vision loss clinically. The exact pathogenesis of RGC loss in DR remains unclear, and many molecules get involved in regulating this process. What is certain, however, is that RGC apoptosis induced by oxidative stress, mitochondrial dysfunction, extracellular glutamate accumulation and molecule expression alterations is the primary mode of death, which occurs in early DR (Oshitari, 2021).

DJ-1, identified as a multifunctional redox-sensitive protein, serves as a molecular chaperone, offering cellular protection against oxidative stress (Sun and Zheng, 2023). The protein’s ability to translocate to the mitochondria and act as an antioxidant under oxidative stress has been well-documented (Taira et al., 2001). In the context of this study, we focused on unraveling the impact of DJ-1 on RGC function and survival. The functional state of RGCs and retinal integrity is often reflected through ERG signals, with studies indicating diminished ERG signals in diabetic mouse models (Lam et al., 2023). Our ERG results concurred, illustrating that hyperglycemia disrupts retinal integrity and impairs visual function. Notably, the absence of DJ-1 further exacerbated these impairments under hyperglycemic conditions. Additionally, the decrease in the amplitude of OPs, indicative of blood circulation in the inner retina (Weber et al., 2008), under hyperglycemia and DJ-1 KO signifies a disturbance in inner retinal blood circulation. While DJ-1 OE resulted in a statistically significant increase in amplitude, the exact implications for inner retinal blood circulation remain unclear and warrant further investigation.

Our study aligns with prior research associating RGC depletion and reduced nerve fiber layer thickness with DR (Lechner et al., 2017; Lai and Lo, 2013). Histological examination using HE staining and RGC counting delineated a neuroretinal landscape in DR marked by increasing RGC loss, retinal disorder and dysfunction. In addition to hyperglycemia, the absence of DJ-1 intensified mitochondrial dysfunction-induced neuroretinal atrophy and RGC loss, emphasizing the critical role of normal mitochondrial homeostasis and ROS scavenging for RGC survival. Confirming expectations, DJ-1 OE mitigated RGC loss and neuroretinal atrophy.

Further investigations into mitochondrial function, ROS production, and apoptosis levels of RGCs revealed that HG culture increased ROS content in R28 cells, a phenomenon alleviated by DJ-1 OE. DJ-1 not only reduced RGC mitochondrial dysfunction induced by HG but also attenuated apoptosis of RGCs by regulating mitochondrial function to prevent oxidative stress damage. Therefore, these results emphasized the vital role of mitochondrial function in RGC apoptosis and determining RGC survival.

In the context of DR, both retrograde axonal transport impairment and glutamate release are considered indirect contributors to RGC loss (Potilinski et al., 2020). Conversely, direct contributors encompass the aberrant expression of neurotrophic factors, growth factors, cytokines, and the accumulation of ROS (Potilinski et al., 2020). ROS, known to reduce the activities of superoxide dismutase (SOD) and other antioxidant molecules (Cecilia et al., 2019), exhibited inhibited expression levels of MnSOD, Catalase, and GPx-1/2 under HG conditions in alignment with our findings. This inhibition, associated with the apoptosis of retinal neurons, including RGCs (Dong et al., 2013; Li et al., 2014), underline the crucial role of oxidative stress in DR.

Mitochondria, acting as both the source and target of ROS, are implicated in mitochondrial dysfunction through interactions with mitochondrial DNA, lipids, and proteins (Miller et al., 2020). The ensuing mitochondrial-mediated apoptosis, an endogenous apoptotic pathway, involves ROS-induced release of Cyto c into the mitochondrial intermembrane, initiating the apoptotic cascade through the activation of cytoplasmic caspase3 (Adebayo et al., 2021). The WB results in our study revealed a decrease in the expression of Bcl-2 under HG conditions, accompanied by increased expression levels of Cyto c, Bax, and cleaved caspase3. Overexpression of DJ-1 countered these effects by upregulating Bcl-2 and antioxidant enzymes such as MnSOD and Catalase. This resulted in an increased Bcl-2/Bax ratio, decreased expression of Bax and Cyto c, highlighting the protective role of DJ-1 against oxidative stress and mitochondrial dysfunction. The stabilization of the anti-apoptosis/pro-apoptosis protein ratio on the mitochondrial membrane and the maintenance of mitochondrial membrane integrity contributed to the reduction in the release of Cyto c and the inhibition of caspase3 activation (Liang et al., 2017).

Our study highlights the potent inhibitory effect of DJ-1 on RGC apoptosis, primarily achieved through the modulation of mitochondrial function and antioxidant effects. However, this protective effect is compromised in the context of DR, where DJ-1 expression is significantly suppressed. Notably, a recent study validating the inhibitory role of DJ-1 in elevated ROS levels and caspase3 expression under oxidative stress in nucleus pulposus cells further elucidates the molecular mechanisms of DJ-1 in the context of DR (Lin et al., 2023).

The sustenance of neural survival and function hinges on the intricate ballet of mitochondrial function, orchestrating the production of cellular adenosine triphosphate (ATP) and calcium storage and buffering (Sheng and Cai, 2012). In the realm of neurodegenerative diseases, disruption of mitochondrial homeostasis and ensuing dysfunction is a well-established hallmark. Our study adds a novel dimension by affirming that HG conditions act as a suppressor of DJ-1 expression, unraveling a cascade effect that disrupts mitochondrial homeostasis and redox balance. This disruption manifests in the accumulation of ROS and the consequent onset of oxidative stress injury.

The authors acknowledged some limitations of this study. We only utilized a type 1 diabetes model, so further research is needed to determine if the conclusions apply to type 2 diabetes as well. Additionally, while the R28 cells used in the *in vitro* model serve as a good substitute for RGCs, validation using primary RGCs would provide more direct evidence. Our findings suggest that DJ-1 has an antioxidant effect and promotes RGC survival under high glucose conditions, making it a potential therapeutic target. However, the underlying molecular regulatory mechanisms require further elucidation. Despite these limitations, this study offers new insights into the treatment of RGC damage in DR and highlights the potential of DJ-1 as a mitochondrial protector and antioxidant.

In summary, our findings position DJ-1 as a promising therapeutic candidate for addressing oxidative stress-related RGC injury in DR. Its robust antioxidant capacity opens avenues for deeper investigations, urging a comprehensive understanding of the intricate interplay among oxidative stress, mitochondrial dysfunction, and senescence. These insights are pivotal for advancing the frontiers of therapeutic interventions aiming to safeguard RGCs in the intricate landscape of DR.

## Data Availability

The original contributions presented in the study are included in the article/Supplementary Material, further inquiries can be directed to the corresponding author.
