# Supplementary figures and images for: DJ-1 regulates mitochondrial function and promotes retinal ganglion cell survival under high glucose-induced oxidative stress
Source: Front Pharmacol. 2024 Sep 11;15:1455439. doi: 10.3389/fphar.2024.1455439 (PMC11422208; doi:10.3389/fphar.2024.1455439)

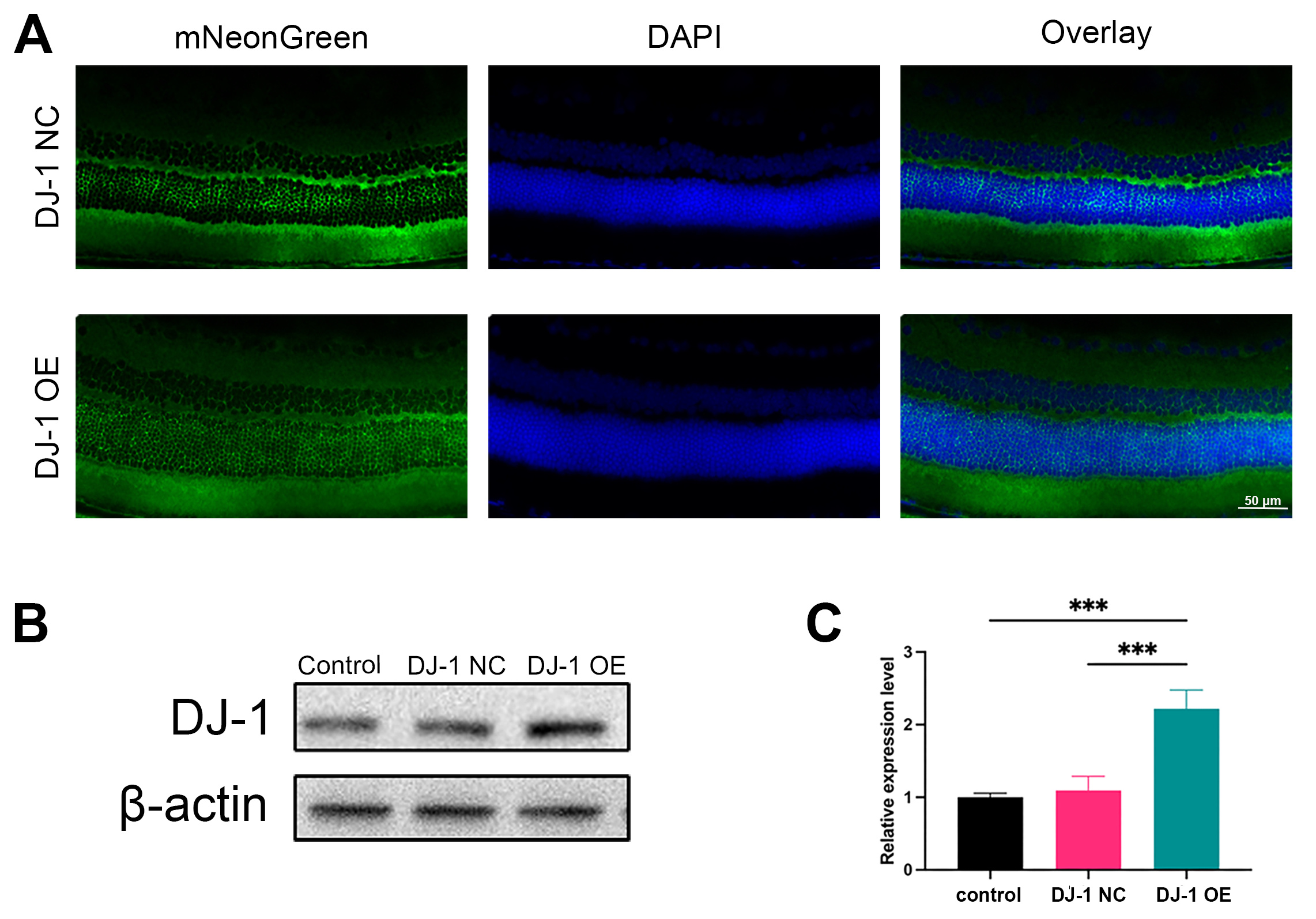

Supplement: Supplementary file 1 [file Image3.TIF]

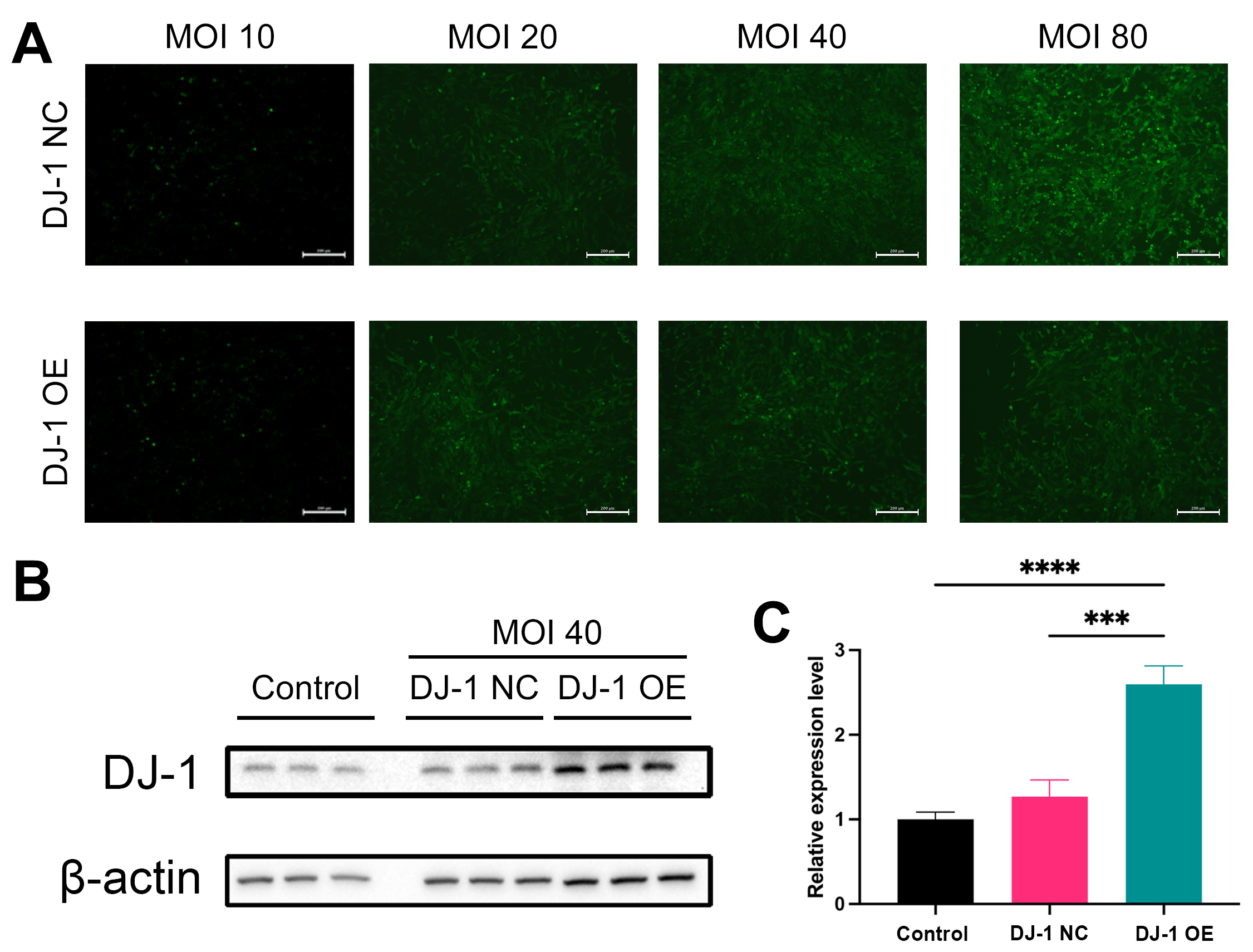

Supplement: Supplementary file 2 [file Image4.TIF]

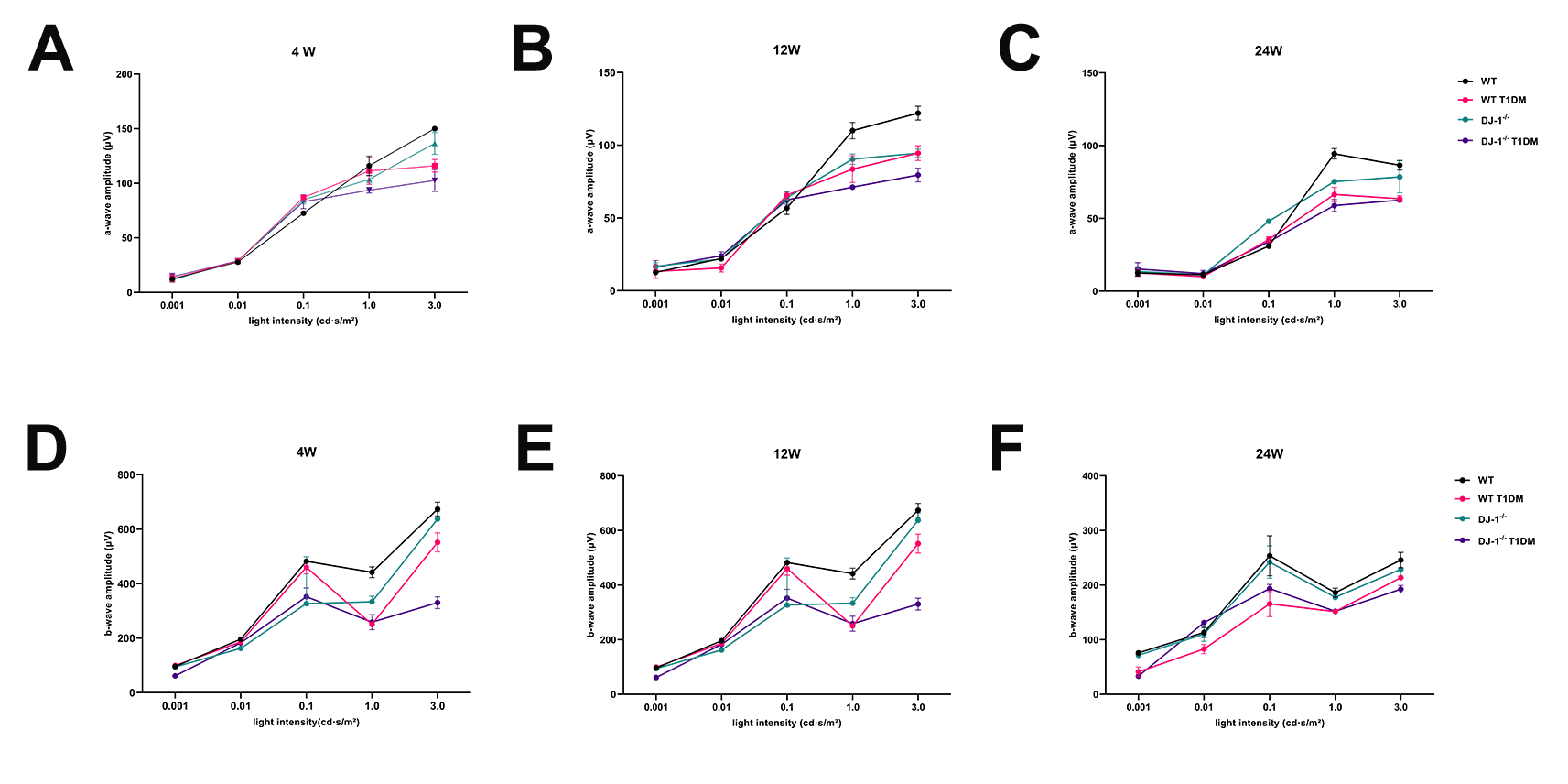

Supplement: Supplementary file 3 [file Image2.TIF]

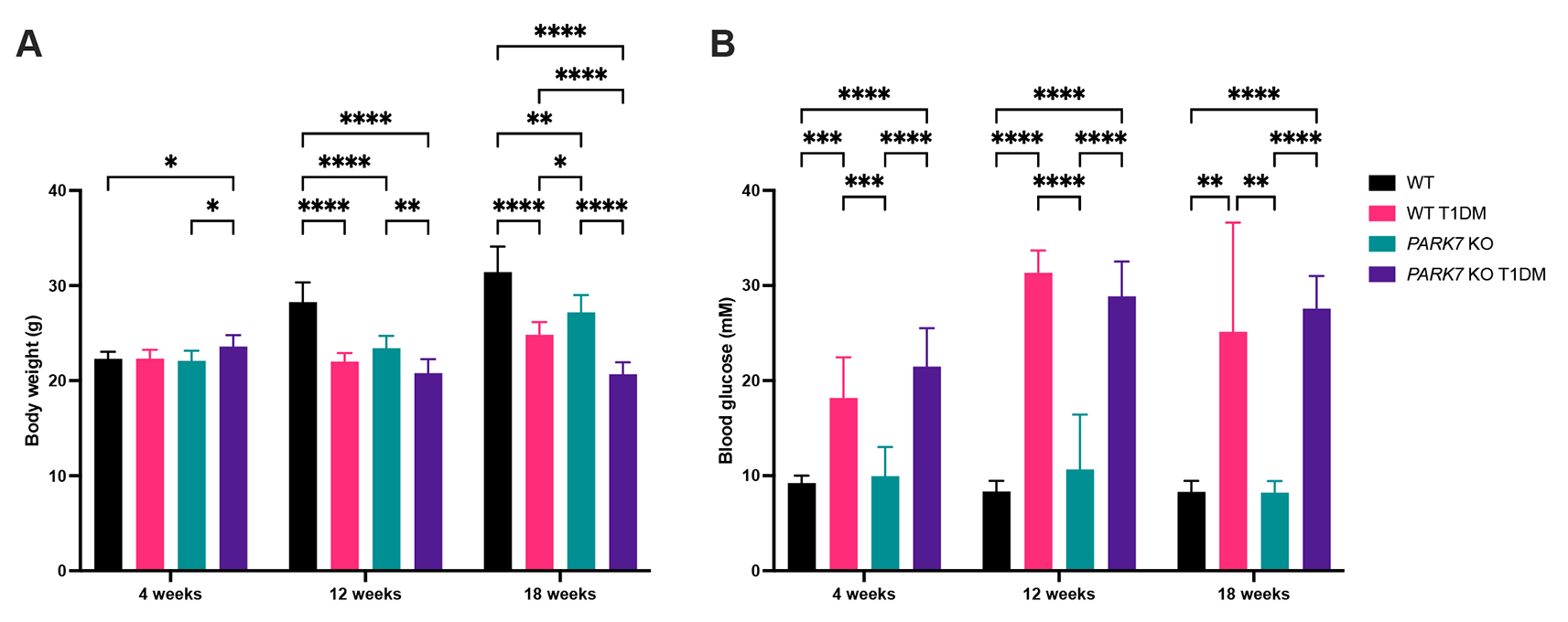

Supplement: Supplementary file 4 [file Image1.TIF]
