# Supplementary material for: DJ-1 regulates mitochondrial function and promotes retinal ganglion cell survival under high glucose-induced oxidative stress
Source: Front Pharmacol. 2024 Sep 11;15:1455439. doi: 10.3389/fphar.2024.1455439 (PMC11422208; doi:10.3389/fphar.2024.1455439)
Supplement: Supplementary file 5 [file DataSheet1.PDF]

Supplementary Table 1

Antibody	Species	Dilution	Company	Catalog #
β III Tubulin	Rabbit	IF 1 μg/mL	Abcam	ab18207
DJ-1	Rabbit	IF 1:200; WB 1:1000	Cell Signaling Technology	5933T
Bax	Rabbit	WB 1:1000	Cell Signaling Technology	14796S
Bcl-2	Rabbit	WB 1:1000	Abcam	ab194583
Catalase	Rabbit	WB 1:1000	Cell Signaling Technology	14097S
MnSOD	Rabbit	WB 1:5000	Abcam	ab13533
GCLC	Rabbit	WB 1:1000	Abcam	ab207777
Cyto c	Rabbit	WB 1:500	Wanleibio	WL02410
GPx-1/2	Rabbit	WB 1:500	Wanleibio	WL02497
Caspase3	Rabbit	WB 1:500	Wanleibio	WL02117
β-actin	Mouse	WB 1:1000	Cell Signaling Technology	3700T
DyLight 488-conjugated goat-anti-rabbit IgG	Goat	IF 1:500	Boster	BA1127
HRP-conjugated goat anti-rabbit secondary antibodies	Goat	WB 1:500	Boster	BA1054
HRP-conjugated goat anti-mouse secondary antibodies	Goat	WB 1:500	Boster	BA1050
